# Electroanatomical mapping after cardiac radioablation for treatment of incessant electrical storm: a case report from the RAVENTA trial

**DOI:** 10.1007/s00066-023-02136-z

**Published:** 2023-09-12

**Authors:** Lena Kaestner, Judit Boda-Heggemann, Hannah Fanslau, Jingyang Xie, Achim Schweikard, Frank A. Giordano, Oliver Blanck, Boris Rudic

**Affiliations:** 1 DKFZ Hector Cancer Institute at the University Medical Center Mannheim, Mannheim, Germany; 2 Department of Radiation Oncology, University Medical Center Mannheim, Medical Faculty Mannheim, University of Heidelberg, Theodor-Kutzer-Ufer 1–3, 68167 Mannheim, Germany; 3 Institute for Robotics and Cognitive Systems, University of Luebeck, Luebeck, Germany; 4 Department of Radiation Oncology, University Medical Center Schleswig-Holstein, Kiel, Germany; 5 I. Department of Medicine: Cardiology, Angiology, Hemostaseology and Intensive Care, University Medical Center Mannheim, Medical Faculty Mannheim, University of Heidelberg, Mannheim, Germany; 6 DZHK (German Centre for Cardiovascular Research) partner site Mannheim, Mannheim, Germany

**Keywords:** Ventricular tachycardia, Stereotactic arrhythmia radioablation, Electrical storm, Electroanatomical mapping, Catheter ablation, Structural heart disease

## Abstract

**Background:**

Electroanatomical mapping (EAM)-guided stereotactic arrhythmia radioablation (STAR) is a novel noninvasive therapy option for patients with monomorphic ventricular tachycardia (VT) refractory to antiarrhythmic drugs and/or urgent catheter ablation (CA). Data on success rates in an emergency situation such as electrical storm (ES) are rare. We present a case of a patient with an initially very poor life expectancy after extensive myocardial infarction with therapy–resistant ES, not amendable for further antiarrhythmic drug therapy, implantable cardioverter-defibrillator implantation, or repeated CA who was introduced to the radiation oncology department for emergency STAR as a bail-out therapy.

**Methods:**

Target volume definition and transfer from EAM to CT were validated and quality assured with a semi-automatic, dedicated visualization tool (CARDIO-RT). Emergency STAR was performed with 25 Gy in the framework of the RAVENTA study. The VT burden gradually decreased after STAR; however, a second VT morphology occurred, which was successfully treated with EAM-guided CA 12 days after STAR.

**Results:**

The second EAM-guided CA showed areas of low voltage in the irradiated segments, indicating a precise targeting and early functional response to STAR. The patient remained free of any VT recurrence or any radiation-related toxicities and in good general condition during the recent follow-up of 18 months.

**Conclusion:**

The case highlights the possible approach, caveats, difficulties, and prognosis of a patient severely affected by therapy-resistant VT in whom CA could not lead to VT suppression. Further studies of putative mechanisms of STAR in the acute and chronic phase of this novel therapy are warranted.

## Introduction

Electrical storm (ES) is a life-threatening cardiac condition defined by the occurrence of three or more episodes of sustained ventricular tachycardia (VT) within 24 h. Recommended treatment options comprise direct current cardioversion, antiarrhythmic drugs, urgent catheter ablation (CA), and anti-arrhythmic surgery [1]. Generally, ES responds poorly to therapy and is associated with a high recurrence rate and mortality. Cardiac radioablation (stereotactic arrhythmia radioablation, STAR) has effectively been used as a bail-out or emergency treatment for patients with ES [2].

Electroanatomical mapping (EAM)-guided STAR is a novel noninvasive therapy option for patients with monomorphic VT refractory or ineligible to antiarrhythmic drugs and/or CA. A special challenge during STAR is the target definition on EAM and the transfer of the clinical target volume (CTV) from the EAM system to the planning computed tomography (CT), which, if performed manually, is highly user dependent [3]. To semi-automate this process, EAM-to-CT transfer tools have been introduced by different research groups, which will probably facilitate precision and efficacy [4, 5]. Since the first case series published by Cuculich et al. in 2017, an increasing number of patients have successfully been treated with predominantly elective STAR [6]. However, data on success rates in the emergency situation such as ES are rare [2]. Additionally, there is an ongoing debate on whether STAR effects predominate in the early or late posttreatment period and whether radiation-induced fibrosis or radiation-induced reprogramming of cardiac conduction contributes to therapy success [7, 8].

We present the case of a patient with therapy-resistant ES, not amendable for further antiarrhythmic drug therapy and CA, who was ultimately treated with emergency STAR in the framework of the RAVENTA study [9]. A secondary monomorphic VT emerged 11 days after STAR and was treated with EAM-guided CA. This case enables an analysis of the short-term effects after STAR with an electrophysiological complete response directly confirmed by repeated EAM. Additionally, the manual transfer of the CTV from EAM to CT was validated and quality assured with a semi-automatic, dedicated STAR visualization tool (CARDIO-RT).

## Case report

A 63-year-old female patient with history of smoking, obesity, diabetes mellitus, and coronary heart disease was admitted with subacute ST elevation due to a posterior wall myocardial infarction caused by a subtotal occlusion of the proximal right coronary artery, which was successfully revascularized by percutaneous coronary intervention (PCI) 14 days before eventually being treated with STAR. Shortly thereafter she developed cardiogenic shock and was intubated. Inotropes were started (dobutamine, levosimendan) for treatment of the acutely decompensated congestive heart failure. Subsequently, the patient developed an ES with frequent episodes of monomorphic VT (220 bpm) treated with external cardioversion. Amiodarone was first introduced, followed by ajmaline; however, the VT recurrence could not be abolished. Another PCI of the circumflex artery and left anterior descending artery was performed 3 days before STAR, without effect on VT suppression. An implantable cardioverter–defibrillator (ICD; Charisma DR, Boston Scientific, Marlborough, MA, USA) was implanted for secondary prevention.

### Pre-STAR EAM during first CA

An EAM was performed, 2 days prior to STAR, with CARTO3 (Biosense Webster, Diamond Bar, CA, USA), revealing a large endocardial scar area in the left ventricle. Extensive CA of late potentials at the inferobasal and midventricular septal portion of the left ventricle was administered, rendering the VT non-inducible by the end of the procedure. A few hours later, VT re-occurred prompting several ICD shocks. Medication with mexiletine and lidocaine was started, together with a percutaneous stellate ganglion block, which suppressed the VT without total abolishment. The 12-lead ECGs of the recurrent VT pattern were analyzed, and segment 2 and segment 1 were identified as possible exit sites of the VT.

### Emergency STAR

**Fig. 1:**
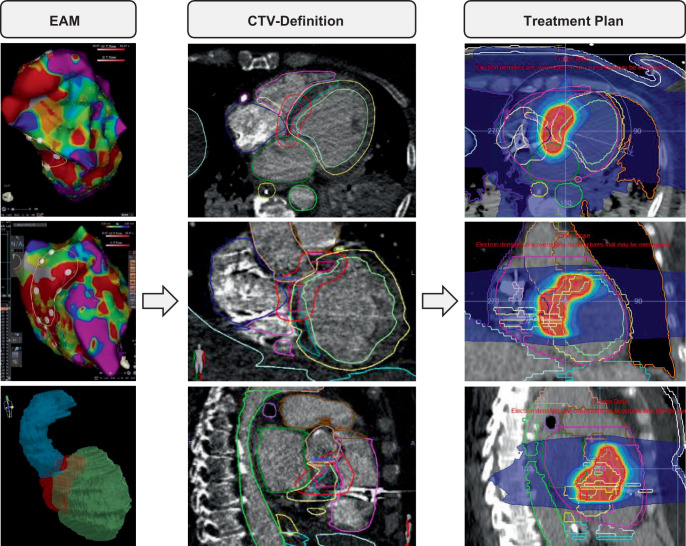
*From the left*: EAM of the left ventricle (superior–inferior and anterior–posterior views, 3D reconstruction; voltage range 0.2–0.8 mV). Defined STAR target (CTV, *pink*; PTV, *red*) after transfer from EAM to the planning CT (transversal, coronal, and sagittal views). Radiotherapy plan dose distribution with a single dose of 25 Gy. *EAM* electroanatomical mapping, *CTV* clinical target volume

As repeated CA was not deemed to be successful, the patient was transferred for emergency STAR after acquiring emergency Institutional Review Board approval in the framework of the RAVENTA study. After performing a planning CT during mechanical ventilation with low tidal volume and high frequency to limit chest motion (Brilliance Big Bore, Philips, Hamburg, Germany), images were registered with an ECG-gated contrast-enhanced CT scan (Velocity Vx, Varian, Palo Alto, CA, USA). Data of the previously performed EAM were used to localize the VT exit site, based on a visual alignment of the presumed origin of VT (Fig. 1). At first, the VT exit site was transferred manually from the EAM to the CT. Additionally, a quality assessment was performed via the semi-automatic CTV transfer tool CARDIO-RT (CARDIO-RT is available free of charge upon request from the authors; [3]). A CTV-to-PTV (planning target volume) margin of 7 mm was applied, and a STAR plan was calculated (Monaco V, Elekta, Stockholm, Sweden) considering the dose and constraint recommendations of the RAVENTA study protocol [10]. A single fraction of 25 Gy prescribed to 95% of the PTV with 6 MV flattening-filter-free (FFF) beams with previous cone-beam CT-based image guidance was delivered on a linear accelerator (Versa HD, Elekta, Stockholm, Sweden). During beam application, mechanical ventilation with low tidal volume and high frequency ventilation was used again to decrease chest excursions and thereby reduce the respiratory motion-based intrafractional error.

### Post-STAR EAM during second CA

**Fig. 2:**
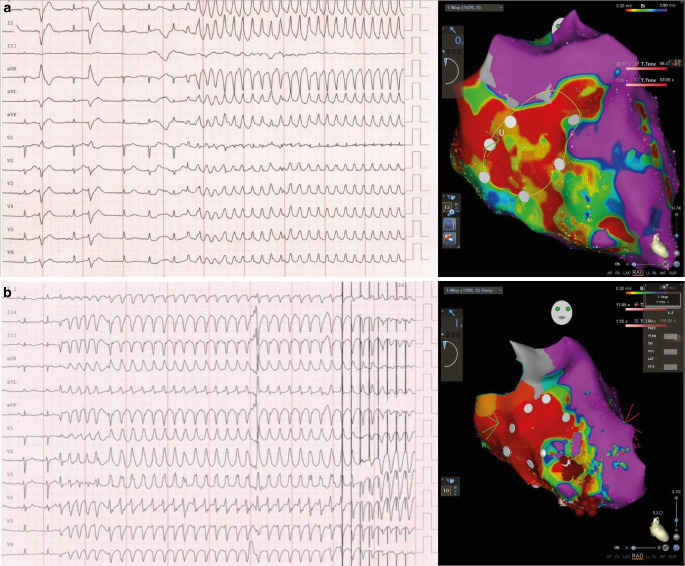
**a** The 12-lead ECGs of the dominant ventricular tachycardia (VT) pattern and right anterior oblique view of electroanatomical mapping (EAM) before stereotactic arrhythmia radioablation (STAR). Possible VT exit sites: segment 1 and 2 (*yellow circle* on EAM). **b** The 12-lead ECGs of the dominant VT pattern after STAR. Possible VT exit sites: segment 9 and 14. Note the extensive area of low voltage (< 0.2 mV) in segments 2 and 1 corresponding to the area previously treated with STAR

After STAR, the VT burden gradually decreased over 4 days. At 5 days after STAR, a second VT morphology occurred repeatedly, now originating from the mid-inferior septum and the septal apex (segments 9 and 14). We did not observe any VT recurrences of the same morphology as the pre-STAR type. Another CA targeting the apex and midventricular septum was performed that successfully treated the second VT pattern. At 10 days after STAR, endocardial EAM showed an extensive area of low voltage (< 0.3 mV) in segments 2 and 1. This area corresponds to the region previously treated with STAR, indicating the precise targeting and early functional response to STAR (Fig. 2).

### Outcome after emergency cardiac STAR and CA

**Fig. 3:**
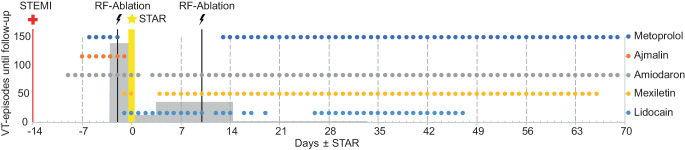
Overview of the antiarrhythmic medication (*dots*) and ventricular tachycardia (*VT*) burden (*gray bars*) from hospital admission until transfer to the rehabilitation clinic. The VT episodes were extracted from the implantable cardioverter–defibrillator (ICDs) implanted on days −3 and 62 (ICD explantation on day 20 due to infection) and ICU monitoring for each follow-up (days 0, 3, 14, 33, 45, 59 after STAR). *STEMI* ST-segment elevation myocardial infarction, *RF* radiofrequency, *STAR* stereotactic arrhythmia radioablation

Antiarrhythmic drug therapy was de-escalated from amiodarone, lidocaine, beta-blockers, and mexiletine to amiodarone and beta-blockers (Fig. 3). During her in-hospital treatment, the patient developed fever and bacteremia with presumed defibrillator lead-associated endocarditis (not related to radiotherapy). The ICD system was successfully extracted, and the patient received antibiotic treatment prior to ICD reinsertion 4 weeks later. Afterwards, the patient was transferred to the rehabilitation clinic and remained in ambulatory care, free of any VT recurrence or any radiation-related toxicities and in good general condition during the recent follow-up of 18 months. Cardiac function stayed stable with moderately reduced left ventricular ejection fraction (42%) and inferior wall akinesia.

## Discussion

We present a case report of a patient with repeated EAM before and after emergency STAR for therapy-refractory ES. In addition to already published case reports and case series [2, 11], this case report presents an analysis of the short-term post-STAR functional effect on EAM and a first impression on quality assessment of manual CTV transfer from EAM to CT with a semi-automated transfer tool.

Emergency STAR was performed on a patient with ES and failure of previous antiarrhythmic medication, ICD implantation, and CA. Krug et al. strongly agree that patients with the clinical constellation of structural heart disease, ICD, recurrent monomorphic VT and ES, and recurrence after CA are eligible for STAR [12]. However, there was no consensus on whether life expectancy < 6 months should be a general contraindication. In this case, the indication for STAR was made despite a poor prognosis, eventually saving the patient’s life. In this way, STAR may prolong life significantly for selected patients with initial poor life expectancy and should be considered as a treatment option.

With STAR, a high-dose radiation of 25 Gy has to be applied in only one treatment fraction. Therefore, precise target definition is crucial for treatment success. Significant challenges occur when the expected target volume needs to be delineated during EAM and transferred to the planning CT. Traditionally, VT ablation is performed in areas of low voltage (0.2–0.8 mV) representing critical isthmus and exit sites of clinical VT, identified with 12-lead ECG. This is usually an iterative and repetitive process with a defined endpoint (non-inducibility of clinical VT). For STAR, a definitive and circumscribed target area needs to be defined by cardiologists. The next crucial step is the transfer of target volume defined in the EAM to the planning CT. Semi-automated tools such as CARDIO-RT used in this case help to compensate manual errors. These tools are currently not commercially available and undergo continuous development and improvement. Interpretation of the target volume depends on a multidisciplinary team approach involving cardiologists and radiation oncologists. Thus, a uniform definition of a target volume has to be standardized in future for further improvement of treatment quality.

In the repeat EAM 10 days after STAR, we noted significant low-voltage areas in segments 1 and 2, which corresponded to the area treated with STAR previously. The mechanisms leading to such a low-voltage area after STAR are not yet fully understood. In the acute and subacute phase after STAR, experimental and clinical studies identified different cellular changes including cellular necrosis and apoptosis, vascular effects, as well as acute mitochondrial damage [13]. Zhang et al. found increases of Na_v_1.5 and Cx43 and concluded that radiation-induced reprogramming of cardiac conduction is a potential treatment strategy [7, 8]. Initial clinical evidence showed changes of ventricular conduction velocity in myocardium subject to 25 Gy or additional ablation modalities [14]; however, all three patients underwent multiple ablation procedures and the time until post-STAR EAM-guided CA ranged from 32 to 395 days. Benali et al. reported on a patient with reduction in bipolar voltages for myocardium receiving ≥ 15 Gy 8 months after STAR [15]. These findings suggest that the observed low-voltage area might be due to changed conduction velocity rather than occurrence of necrosis. This is further supported by the observation that a radiation dose of 25 Gy does not seem to be sufficient to create necrosis in myocytes [16].

Since areas of low voltage assessed in EAM can result from, e.g., changes in conduction velocity, necrosis, as well as fibrosis, we cannot exclude the possibility that, apart from STAR, the documented low-voltage area could also be a result of the previous myocardial infarction. However, the exact location corresponding to targeted areas 1 and 2 and its sharp delineation are favorably interpreted as an effect of STAR rather than acute fibrosis due to recent myocardial infarction.

## Conclusion

Stereotactic arrhythmia radioablation (STAR) is an expedient therapy alternative even in severe cardiac conditions such as electrical storm (ES) in cases of failed or ineffective catheter ablation (CA). Our case highlights the possible approach, caveats, difficulties, and prognosis in a patient severely affected by therapy-resistant ventricular tachycardia (VT) in whom CA could not lead to VT suppression. Here, STAR led to a significant circumscribed low-voltage area captured during electroanatomical mapping 12 days after STAR. Further studies of putative mechanisms of STAR in the acute and chronic phase of this novel therapy are warranted.
